# Risk chain identification of single-vehicle accidents considering multi-risk factors coupling effect

**DOI:** 10.1371/journal.pone.0302216

**Published:** 2024-05-23

**Authors:** Fangyuan Li, Xia Wang, Zenglei Feng, Jian Wang, Mengdi Li, Kun JIANG, Changli ZHAO

**Affiliations:** 1 School of Automobile Engineering, Shandong Jiaotong University, Jinan, China; 2 School of Transportation, Shandong University of Engineering and Vocational Technology, Jinan, China; Southwest Jiaotong University, CHINA

## Abstract

The real-time monitoring on the risk status of the vehicle and its driver can provide the assistance for the early detection and blocking control of single-vehicle accidents. However, complex risk coupling relationship is one of the main features of single-vehicle accidents with high mortality rate. On the basis of investigating the coupling effect among multi-risk factors and establishing a safety management database throughout the life cycle of vehicles, single-vehicle driving risk network (SVDRN) with a three-level threshold was developed, and its topology features were analyzed to assessment the importance of nodes. To avoid the one-sidedness of single indicator, the multi-attribute comprehensive evaluation model was applied to measure the comprehensive effect of characteristic indicators for nodes importance. A algorithm for real-time monitoring of vehicle driving risk status was proposed to identify key risk chains. The result revealed that improper operation, speeding, loss of vehicle control and inefficient driver management were the sequence of top four risk factors in the comprehensive evaluation result of nodes importance (mean value = 0.185, SD = 0.119). There were minor differences of 0.017 in the node importance among environmental factors, among which non-standard road alignment had the larger value. The improper operation and non-standard road alignment were the highest combination correlation of factors affecting road safety, with the support of 51.81% and the confidence of 69.35%. This identification algorithm of key risk chains that combines node importance and its risk state threshold can effectively determine the high-frequency risk transmission paths and risk factors through multi-vehicle test, providing a basis for centralization management of transport enterprises.

## Introduction

The total mileage of highway, use of civilian vehicles and number of motor vehicle drivers in China have gone up by 2.71 times, 15.99 times and 4.97 times over the past 20 years, respectively [1]. The continued improvement in facilities and level of traffic management could not keep up with the dramatically increase in traffic demand, resulting in lots of safety concerns. In 2021, more than 273,000 road traffic accidents and 62,218 deaths were reported in China, up by 38.76 percent and 6.32 percent respectively over that of 2014 [2]. A path-breaking investigation was conducted by Heinrich (1931), and found that the proportion relationship of serious injury accidents slight injury accidents and no injury accidents in a mechanical manufacturing company was counted as 1: 29: 300. In other words, when multiple risk factors interact with each other, a series of errors can occur, which can easily lead to major accidents. Similar findings were found in road traffic accidents, subway construction accidents, chemical accidents and coal mine accidents [3–6]. From 2010 to 2020, there were 151 major road traffic accidents with 10+ fatalities, more than half of which was single-vehicle accidents. Commercial vehicle accidents accounted for more than 80% of the total accidents.

A increasing body of research has investigated the contributing factors of accident frequency and injury severity for single- and multi-vehicle accidents separately [7–9]. The results have shown that the differences in the mechanisms and contributing factors between single- and multi-vehicle accidents are significant. In numerous studies on the contributing factors of single-vehicle accidents, human factor has been recognized as the principal factor influencing the incidence and severity of single-vehicle accidents [10]. Furthermore, a certain number of studies has focused on the impact of vehicle factors, environmental factors and risk factors related to the enterprise management for motor vehicle accidents. Moreover, each major accident show complex risk coupling relationship, resulting from interactions among multiple factors. Many researchers have paid extensive attention to evaluate the joint effect of multiple factors, such as weather and lighting conditions [11], traffic lane and shoulder widths [12], vehicle type and light condition [13], environment, traffic, and population characteristics [14], economic stress and urbanization [15], combination of road infrastructure elements [16], enterprise administration, external environment and driver attitudes to traffic safety [17].

At present, data-driven statistical model and graph theory are the main methods to analyze the coupling relationship among accident risk factors. The former includes random parameter regression model [18, 19], Bayesian model [20], shrinkage regression model [21], finite mixture model [22] and Markov transformation model [23], etc. Data-driven statistical methods are difficult for users to find out the evolution process of risk factors in essence. Even though the interactive impacts of risk factors have been extensively studied by using the above model, these studies had neglected chain reactions between accident risk factors. The latter graphically displays the relationship of the risk factors, such as event tree analysis [24], SDG model [4] and petri nets [25], etc. Comparatively speaking, SDG model not only reveals the risk transmission path, but also comprehensively explains the occurrence regularity of accidents [26], so that it can provide specific analysis on the characteristics of network structure. Compared with general accidents, the joint effect of multiple contributing factors is more complex in the major accident network. The coupling evolution mechanism of multiple factors has not been fully elucidated to date.

Before a major accident occurs, the fluctuation on risk degree of paths will become obvious. If the key risk transmission path can be identified, the accident frequency and loss will be greatly shortened by timely extraction of high risk nodes. In this context, scholars in different fields have proposed path recognition methods based on graph theory to monitor the risk degree of transmission paths. Wu et al. (2021) [27] examined in their study that the combination of multi-threshold method, trend fitting, and SDG model to conduct online monitoring and risk detection for the nuclear power plants was superior in speed and precision to the conventional SDG method. Zhang et al. [28] proposed a deep-first search strategy to identify the evolutionary path of chain faults in power grid, on the basis of the combination of knowledge graph and machine learning method to judge time sequence characteristics. In addition, dijkstra’s algorithm [29], and ant colony algorithm [30] were also applied to the identification of key risk transmission paths of various accidents. So far, the complex mechanism of road traffic accidents has not been revealed.

The goal of this study is to map the interactive effect of accident influencing factors from four aspects, namely human, vehicle, environment and enterprise administration. To that end, the investigation reports of single-vehicle accidents with 10+ fatalities and enterprise safety management database throughout the life cycle of vehicles were statistically analyzed. The main contributions of this study are as follows: (1) identify two categories of relationships inclusive of inter-relationships between various risk factors, intra-relationships between risk factors and accidents within the network-based approach in the SVDRN. (2) propose the algorithm of risk chain identification to identify key risk transmission path, basing on a combination of node importance and its risk state threshold in the SVDRN.

## Methodology

### Analysis procedures

The backward inference method along the risk transmission paths was used to extract potential risk factors in all aspects of the vehicle’s daily operation, starting from the result of the accident. The SVDRN was established based on the SDG model, in which the risk factors of single-vehicle accidents and their connectivity were taken as the network nodes and directed edges respectively. The topological structure of SVDRN was analyzed by using five indicators, including degree centrality, eigenvector centrality, closeness centrality, betweenness centrality and clustering coefficient. Considering the associated impact of the importance nodes and variable risk intensity in the SVDRN, this paper proposed a method for identifying key risk transmission path to real-time monitor the risk status of vehicle driving.

### Data collection

Compared with minor accidents, the investigation of major single-vehicle accidents with 10+ fatalities is more thorough and comprehensive in China. According to Decree No. 493 of the State Council [31], the accident investigation group established by the people’s government of province where the accident occurred is responsible for writing the accident investigation report. The data of major accident needs to contain the upstream and downstream information of the accident, so that the causal relationship of accident can be systematically sorted out. Major single-vehicle accidents at the number of 83 from 2010 to 2020 were analyzed in this paper, and types of accidents at number of 6 were divided as follows: rollover (marked as R1), plunging into water or a ditch (R2), running over pedestrians (R3), fire (R4), natural disaster (R5), collision with fixed objects or parked vehicle (R6). The explanation of risk factors in the SVDRN is depicted in Table 1.

**Table 1:** Description of risk factors in the SVDRN.

Type	Risk factors (No.)	Definition / Explanation
Human factor	driving without a license (F1)	It includes invalid driving license, inconsistency with quasi-driving type or not obtaining driving license.
	speeding (F2)	Decree No.163 of the Ministry of Public Security of the P.R.C. [32] imposes a differential fine and adding points to your driver’s license, according to the overspeed ratio of your vehicle.
	violating the traffic rules (F3)	Decree No. 81 of the President of the P.R.C. [33] illustrates 26 types of violating the traffic rules, such as running the red light, reverse driving of motor vehicles, etc.
	fatigue driving (F4)	Article 62 of order No.405 of the State Council of the P.R.C. [34] stipulates for continuous driving duration and rest time.
	drunk driving (F5)	GB19522-2010 [35] indicates the classification thresholds for blood alcohol concentration of vehicle driver.
	risky driving behavior (F6)	The AI-based face-recognition technology is employed to identify dangerous driving behaviors while driving, such as playing mobile phone, smoking, hands-off driving and not wearing seat belt, etc.
	improper operation (F7)	making an incorrect operation when a driver encounters an emergency. The driver’s improper skill behavior consists of coasting in neutral, hard acceleration and deceleration, hard braking, irregular turning, etc. The comprehensive evaluation is conducted according to the accident frequency in the past three years and the real-time monitoring of driver skill behavior. The vehicle body CAN-bus is used to monitor the driver’s skill behaviors, such as the variation of accelerator, speed and brake, etc.
	negligent observation (F8)	JT/T883-2014 [36] points out the components of commercial vehicle driving dangerous warning system, including forward collision warning system, lane departure warning system and etc.
	driving in the unapproved line (F9)	GPS dynamic monitoring platform of transport enterprise can monitor whether the vehicle deviates from the planned route.
Vehicle factor	interference from the unsafe behavior of other vehicles (F10)	Other vehicle’s dangerous behaviors compels the vehicle to take emergency measures, thereby inducing an accident.
	hidden danger of vehicle (F11)	Safety components are faulty, such as the steering system or braking system.
	low tire pressure (F12)	The data is obtained from tire pressure monitoring system, according to GB 26149–2017 [37] and JT/T1429-2022 [38].
	overload (F13)	Article 92 of Decree No. 81 of the President of the P.R.C. [4] proposes the penalty for passenger vehicles carrying more than the maximum passenger capacity and freight vehicles carrying more than rated load mass.
	Illegally carrying passengers (F14)	carrying passengers in the compartment of a freight vehicle or a tricycle.
	no license plate (F15)	A legal license plate has not obtained.
	loss of vehicle control (F16)	The accidents cannot be prevented, even with appropriate interventions.
Environmental factor	severe weather (E1)	The classification standard of rainfall levels in different time periods and visibility grade are respectively explained according to GB/T28592-2012 [39] and Decree No. 81 of the President of the P.R.C. [4].
	inadequate road infrastructure (E2)	JTG D81-2017 [40] puts forward the technical requirements of road facilities. Failure to meet design requirements, lack of road infrastructure and unrepaired damage are the main phenomenons in road traffic accidents.
	non-standard road alignment (E3)	JTG D20-2017 [41] illustrates standard for road gradient and the curvature radius of road.
	unfavorable terrain (E4)	The road segment in the process of vehicle driving is bridge, tunnel, road along a mountain or near a ditch.
Management factor	vehicle ownership (M1)	It includes three forms: enterprise-owned vehicle, private vehicle attached to a transport enterprise and contract management on transport line.
	failed vehicle dynamic monitoring (M2)	It is measured by the GPS offline rate of the commercial vehicle.
	inefficient driver management (M3)	Decree No.18 of MOT of the P.R.C. [42] explains the cycle and training time of continuing education for road transport drivers.
	negligent vehicle technical management (M4)	The data of vehicle technical management includes pre-trip vehicle inspection, maintenance records, vehicle annual inspection, etc.

### SDG model for single vehicle driving risk network

**Fig 1:**
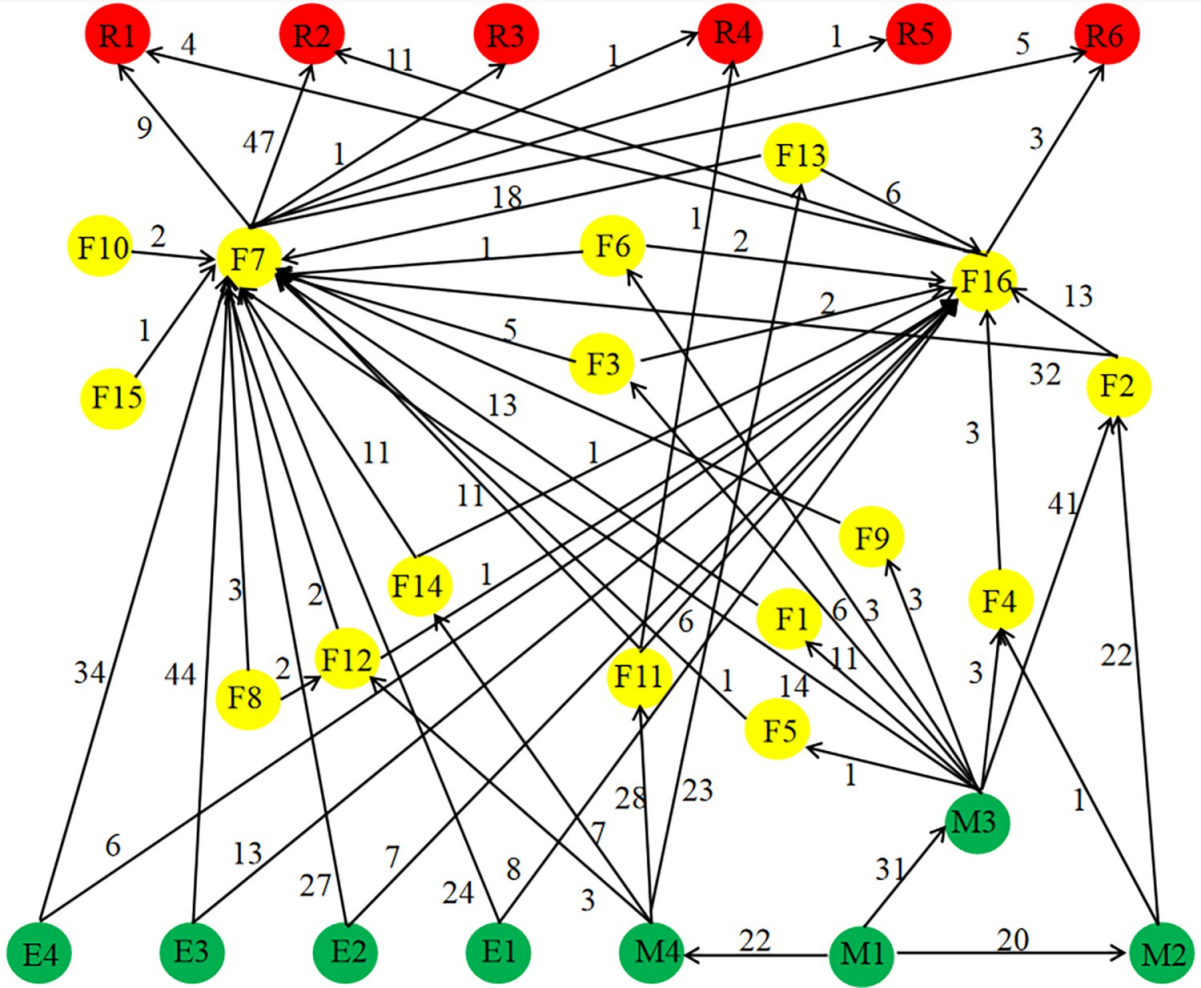
The SDG model for SVDRN.

The adjacency matrix *M* was defined, whose element *a*_*ij*_ was the correlation between node *i* and node *j*. The value of *a*_*ij*_ was equal to 0, when node *i* was not associated with node *j* or the value of *i* was the same as *j*. Otherwise, the value of *a*_*ij*_ was equal to 1. Considering the one-way evolution characteristic of accident chain, the SVDRN was a directed graph corresponding to an asymmetric matrix. According to mutual relations of risk factors, the structure diagram of network node based upon the SDG technology is constructed, as shown in Fig 1. The weight of SVDRN represents the occurrence number of two adjacent nodes in the total sample of accidents.

### Topological features of the SDG model

In order to estimate whether the network has the features of complexity, scale-free and small world, topology parameters need to be calculated, including degree distribution, clustering coefficient and closeness centrality of the network [43]. The more number of sides connected to node *i* is, the larger the degree of node *i* is, explaining that this node is closer to the center of the network, and susceptible to the interaction of neighbor nodes. The degree of a node contains two types in a directed network: in-degree and out-degree, so the total degree value of this node is the sum of both values. Take node M1 in Fig 1 as an example, none of the nodes points to M1, so the in-degree value of M1 is 0. There are three nodes that flow out from M1, including M2, M3, and M4. The out-degree value of M1 is 73, which is equal to the sum of the occurrence number for all the accident samples between M1 and each of three nodes.

The clustering coefficient *C*_*i*_ is the probability that any two neighbors connected to node *i* are also neighbors to each other in complex network theory. The calculation expression of *C*_*i*_ is as follows:

$$C_{i}=\frac{2N_{i}}{k_{i}(k_{i}-1)}$$ (1)

where, *k*_*i*_ is the sum total of sides connected to node *i*, and *N*_*i*_ is the sum total of sides between neighbor nodes connected to node *i*.

Betweenness centrality *BC*_*i*_ points out that a core node should appear on multiple shortest paths of other nodes. The stronger the transmission capacity of node *i* is, the higher the value of *BC*_*i*_ is. The betweenness centrality of node *i* can be obtained as [44]:

$$BC_{i}=\sum_{j}^{n}\sum_{m}^{n}\frac{P_{jm}(i)}{P_{jm}},\;i\neq j\neq m\;and\;i\langle j$$ (2)

where, *P*_*j*m_ is the sum total of shortest paths between node *j* and node *m*, *P*_*jm*_(*i*) is the sum total of shortest paths through node *i*, node *j* and node *m* simultaneously, and *n* is the sum total of nodes in the SVDRN.

Closeness centrality describes the proximity effect of any two nodes in the risk network. *S*_*ij*_ is used to represent the amount of shortest paths from node *i* to all other nodes in the SVDRN. The calculation formulas of closeness centrality for node *i* is defined as [45]:

$$CC_{i}=\frac{1}{\sum_{j=1}^{n}S_{ij}}$$ (3)

The basic thought of eigenvector centrality is that the node importance is dependent on the amount of its neighbors (i.e., degree distribution), as well as the importance of its neighbor nodes. The more important the neighbors connected to a node are, the higher the numerical value of eigenvector centrality is. Suppose *x*_*i*_ represents the importance of node *i*, the eigenvector centrality *EC*_*i*_ can be expressed as in Eq (4). When the steady state is reached after many iterations, it can be written in Eq (5).

$$EC_{i}=x_{i}=c\sum_{j=1}^{n}\;a_{ij}x_{j}$$ (4)

x=cMx (5)

where, *c* is the proportionality constant, and equal to the inverse of the principal eigenvalue of matrix *M*; *x* is the column vector of (*x*_1,_ *x*_2,_ …,*x*_*n*_)^T^.

### Comprehensive evaluation of node importance in the risk network

In view of the limitation of single indicator on evaluation of node importance in practical application, the above-mentioned five indicators were used to construct the multi-attribute comprehensive evaluation model of node importance for SVDRN. The indicator weight of the comprehensive evaluation model was computed by the average weighted standardized matrix, and the value of comprehensive evaluation of node importance was obtained by an ideal scheme.

It was assumed that each node has characteristic indicators at number of *s*, then the *j*th indicator of the *i*th node was defined as *T* = *w*_*i*_(*q*_*j*_), among which the value of *i* ranges from 1 to *n*, and the value of *j* ranges from 1 to *s*. Due to the different dimensions of indicators, the matrix was normalized to establish a multi-attribute matrix of nodes, and carried out by Eqs (6)–(8). The conventional practice is to assign weight for each indicator of evaluation model according to experience. If it is difficult to find reasonable values, the average method of weighting normalized matrix is commonly used [46, 47]. The normalized matrix is denoted as *A* = (*r*_*ij*_)_*n*×*s*_. *K*_*i*_ in Eq (9) represents the comprehensive importance of node *i* for SVDRN. An ideal scheme was adopted to evaluate the value of *K*_*i*_, and the calculation formula of *K*_*i*_ was shown in Eq (9). The values of $U_{i}^{-}$ and $U_{i}^{+}$ are calculated by the euclidean norm in Eqs (10) and (11).

$$r_{ij}=\frac{w_{i}(q_{j})-w_{i}{\left(q_{j}\right)}^{\min}}{w_{i}{\left(q_{j}\right)}^{\max}-w_{i}{\left(q_{j}\right)}^{\min}}$$ (6)

$$w_{i}{\left(q_{j}\right)}^{\max}=\max(w_{i}(q_{j}))$$ (7)

$$w_{i}{\left(q_{j}\right)}^{m\mathrm{in}}=\min(w_{i}(q_{j}))$$ (8)

$$K_{i}=\frac{U_{i}^{-}}{U_{i}^{-}+U_{i}^{+}}$$ (9)

$$U_{i}^{-}={\left\|r_{ij}-r_{j}^{\min}\right\|}_{2}$$ (10)

$$U_{i}^{+}={\left\|r_{ij}-r_{j}^{\max}\right\|}_{2}$$ (11)

where, $r_{j}^{\max}$ and $r_{j}^{m\mathrm{in}}$ represent the highest and the lowest value in the column *j* of matrix *A* respectively.

### Real-time monitoring of risk status with three-level threshold

“Management Measures for the Evaluation of Safety Production Standardization Construction in Transport Enterprises” is issued by Ministry of Transport of China in 2016, urging transport enterprises to establish a technical management database throughout the life cycle of vehicles. The purpose of above regulation is to facilitate the risk evaluation and supervision of vehicle driving safety. Table 2 shows the hierarchical thresholds of risk status for network nodes. The status of evaluation indicators for all the risk factors was determined by the three-level threshold, namely H (high risk), M (medium risk) and N(normal). The values of three-level threshold were 0.9, 0.5 and 0.1 respectively for subsequent quantitative processing. Each risk factor had at least one and at most three evaluation indicators. For the threshold of a factor with multiple evaluation indicators, the threshold of the factor was equal to 0.1, when the risk status of all the evaluation indicators were normal. The threshold of the factor was equal to 0.9, when one or more indicators were at high risk. The threshold of the factor was equal to 0.75, when more than two evaluation indicators were at medium risk. The node data of SVDRN indicated in Table 2 had both real-time dynamic and static data.

**Table 2:** Hierarchical thresholds of risk status for network nodes.

Risk Factors	Evaluation indicators	Three-level threshold		
		Normal	Medium risk	High risk
F1	With or without driver’s license	obtained in accordance with law	invalid driver’s license or inconsistency with quasi-driving type	without
F2	Ratio of speeding, %	≤10	(10, 50)	≥50
F3	Vehicle violation record (driver’s license points per year)	0~3	4~6	7~12
F4	State of fatigue warning system	low	medium	high
	Driver’s continuous driving duration, hour	≤4	(4, 6)	≥6
F5	Driver’s blood alcohol concentration, mg	<20	[20, 80)	≥80
F6	Warning state of bad driving behavior	low	medium	high
F7	Number of accidents caused by improper operation in the past three years	0	1	≥2
	Risk state of driver skill behavior through vehicle body CAN-bus	low	medium	high
F8	State of forward collision warning system	low	medium	high
	State of lane departure warning system	low	medium	high
F9	Whether to deviate from the planned route	no	yes	——
F10	Accidents black spots	no	yes	——
F11	Correction rate of hidden danger investigation for vehicle safety components, %	100	[90, 100)	<90
	Real-time state of vehicle self-check	minor fault	general fault	serious fault
F12	Tire pressure, Kpa (P_rec_ is recommended vehicle pressure)	> 75% *P_rec_	[75%*P_rec_, 50%*P_rec_)	≥50%*P_rec_
F13	Overload ratio (passenger vehicle/freight vehicle), %	0/0	(0, 20) / (0, 30)	≥ 20 / ≥ 30
F14	Illegally carrying passengers	no	below approved load mass	above approved load mass
F15	With or without license plate	obtained in accordance with law	duplicating other vehicle	without
F16	Body state—ESP	ineffectiveness	at work	——
E1	Precipitation (12 hours/ 24 hours), mm	<4.9 / <9.9	[5,30) / [10,50)	≥30 / ≥50
	Snowfall(12 hours/ 24 hours), mm	<0.9 / <2.4	[1, 6) / [2.5, 10)	≥6 / ≥10
	Visibility, m	≥200	(50, 200)	≤50
E2	Road infrastructure condition	complete and unimpeded	occupying-road construction	Lack of road infrastructure
E3	Road longitudinal slope, %	(0,3]	(3, 5]	≥5
	Curvature radius of road, m	≥1000	(1000, 450]	<450
E4	Terrain	general road	general bridge or tunnel	road along a high mountain or near a deep ditch
M1	Ownership of vehicle	enterprise owned vehicle	periodic management except owned vehicle	non-compulsory management except owned vehicle
M2	GPS on-line rate, %	≥95	[90, 95)	<90
M3	Driver training time and examination results	≥16 class hours per year and pass the test	<16 class hours per year and pass the test	<16 class hours per year or fail the test
M4	Implementation rate of vehicle safety daily management, %	100	[90,100)	<90

The active safety intelligent prevention and control system of road transport enterprises comes with the increasingly high requirements for vehicle safety, and it is currently applied at a very high rate, especially for “two passengers and one danger” vehicles. Specifically, “two passengers and one danger” vehicles refer to line buses with the third-class or above, tourist chartered buses and road special vehicles carrying hazardous chemical, civil explosive, fireworks and firecrackers [48]. Generally speaking, the intelligent prevention and control system of vehicle active safety is composed of driving records, satellite navigation system, safety assistance driving system, driver behavior monitoring, etc. The real-time monitoring on drivers’ driving behaviors, vehicle driving environment and overloading of vehicle is carried out by using the system, so as to accomplish the warning of driving safety hazards.

### Algorithm for identifying key risk chains

**Fig 2:**
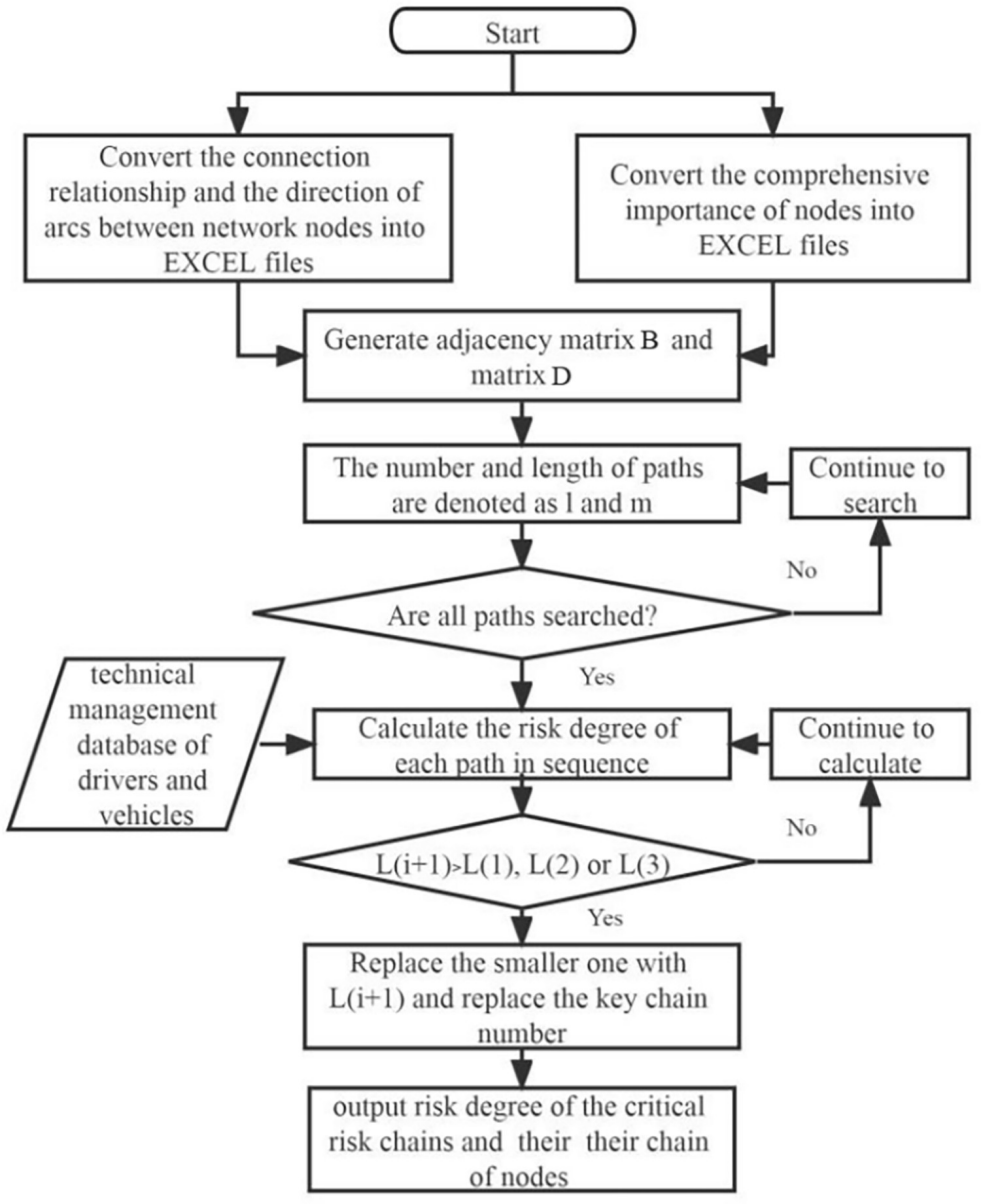
Flow chart of identification algorithm for key risk chain.

The algorithm of risk chain identification for SVDRN was divided into two stages: network structure scanning and the identification of key risk chain. After obtaining the comprehensive importance of each node in the SVDRN, adjacency matrix *B* was set to store the connection relationship and the direction of the arc among network nodes, and matrix *D* was used to store the comprehensive importance of nodes, both of which was based on ergodic search algorithm [49]. The first step in the second stage was to read data from technical management database, so as to record the threshold of risk status for network nodes. The reverse search of SVDRN along the directed arc was carried out according to the path number. The node risk degree was defined as the product of the node’s comprehensive importance and its risk state threshold. The risk degree of path *i* was the product of the risk degree of all nodes on the path, marked as *L(i)*. The path set with top 3 risk degrees was obtained, and all nodes of the key risk chains were output in sequence. Fig 2 illustrates the steps of identification algorithm for key risk chains.

## Result discussion

### Analysis result of the network topology

**Fig 3:**
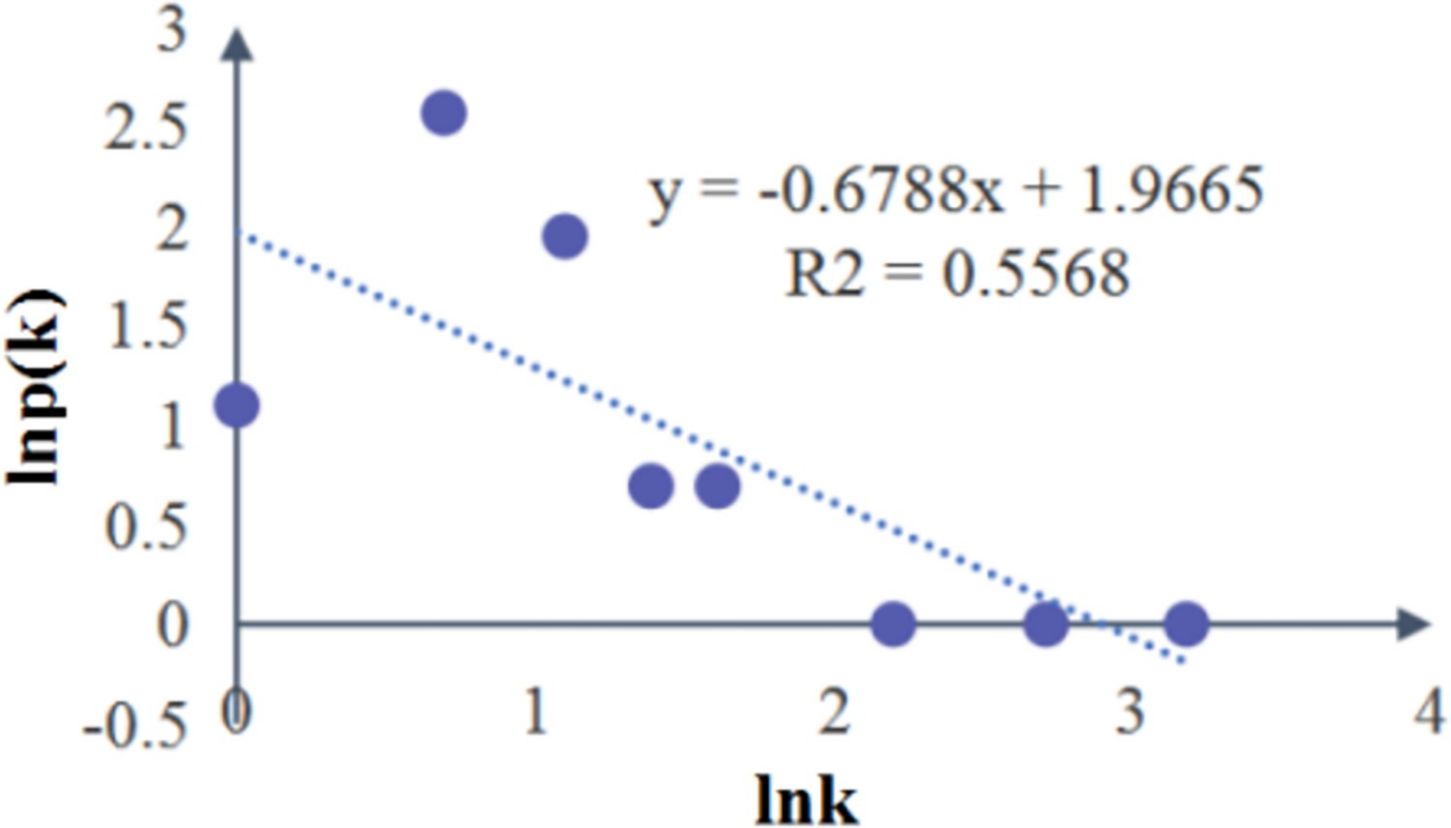
Degree distribution of SVDRN.

As can be shown from Fig 1, there were 30 nodes and 42 edges in the SVDRN, with an mean network density of 0.731 and a standard deviation of 4.155. The steps of all paths in the SVDRN were less than or equal to 4, and the average path length was 2.413. As a matter of fact, most real risk networks have small average path length, which leads to the implications of small world [4]. The average path length is inversely correlated with efficiency of risk transmission. Moreover, the average value of clustering coefficient in the SVDRN was 0.246, indicating that the overall clustering level was more than that of the local. In other words, there were no small groups within the SVDRN. Although the sum total of nodes in the SVDRN was not so large, the coupling relationship from the root node to the leaf node was complex. For example, the number of possible paths from M1 to R6 was 22. Therefore, the structure of SVDRN conformed to the property of complexity and small world. The degree distribution of SVDRN was fitted as a straight line with negative slope, as shown in Fig 3. Y-axis was the natural logarithm of frequency in the Fig 3, and X-axis was natural logarithm of degree value. It had the approximate fit *y* = -0.6788*x* + 1.9665 with *R*^2^ = 0.5568. The nodes with low degree value accounted for the vast majority in a scale-free network, while the nodes with high degree value were only very few [50]. The result denoted that the cumulative distribution of node degree within the SVDRN conformed to a power-law distribution, hence the SVDRN was scale-free.

**Fig 4:**
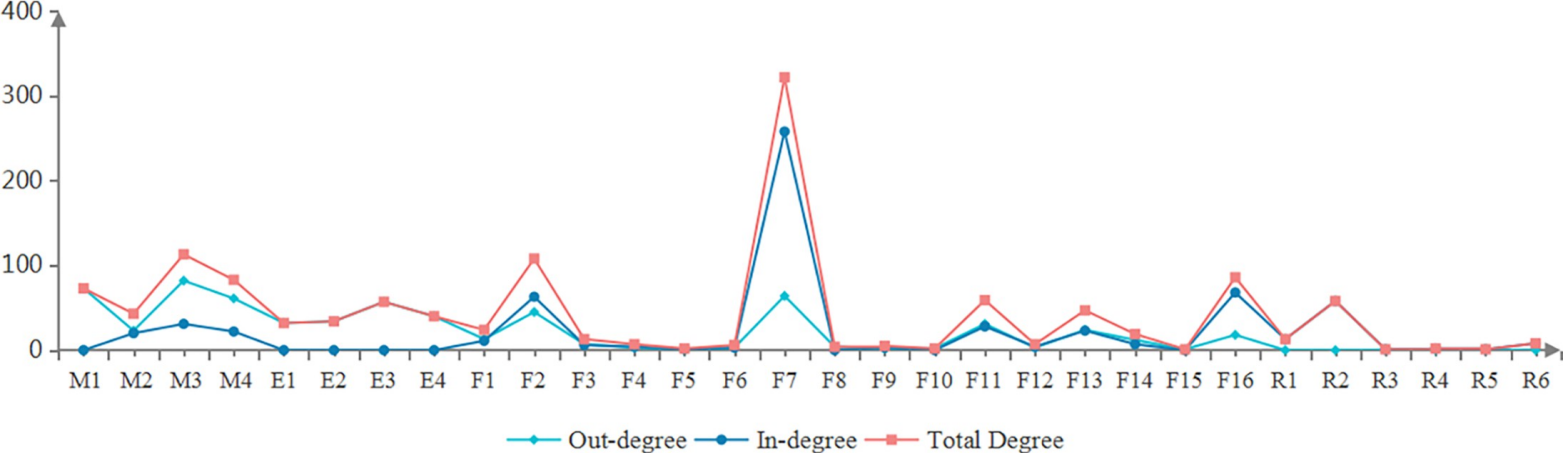
The degree distribution of nodes in the SVDRN.

The degree distribution of 30 nodes in the SVDRN is displayed in Fig 4, which the mean total degree is 13.103. The four nodes of F7 (improper operation), F2(speeding), M3 (inefficient driver management) and F16 (loss of vehicle control) had a higher total degree in the network. The node of F7 had the highest in-degree of 258, indicating that F7 was in a core position in the SVDRN and had a strong correlation with other nodes. The in-degree of F7 was extremely high due to their parent nodes at the number of 17. An accident due to improper operation occurred, involving 1 to 4 parent nodes. The node of M3 was the highest node of the out-degree value, with the value of 82. Measured by the annual training time and assessment results of drivers, it indicated that the driver’s education and awareness levels were the main factors inducing accidents. In addition, the node of F16 was the second highest in-degree with the value at 68, indicating the running state of the vehicle body. Whether ESP works or not was applied to measure the body stability. The node of F2 belonged to the intermediate node, and its total degree value was relatively high. Accordingly, speeding played a important the role of bridge in vehicle driving safety.

**Fig 5:**
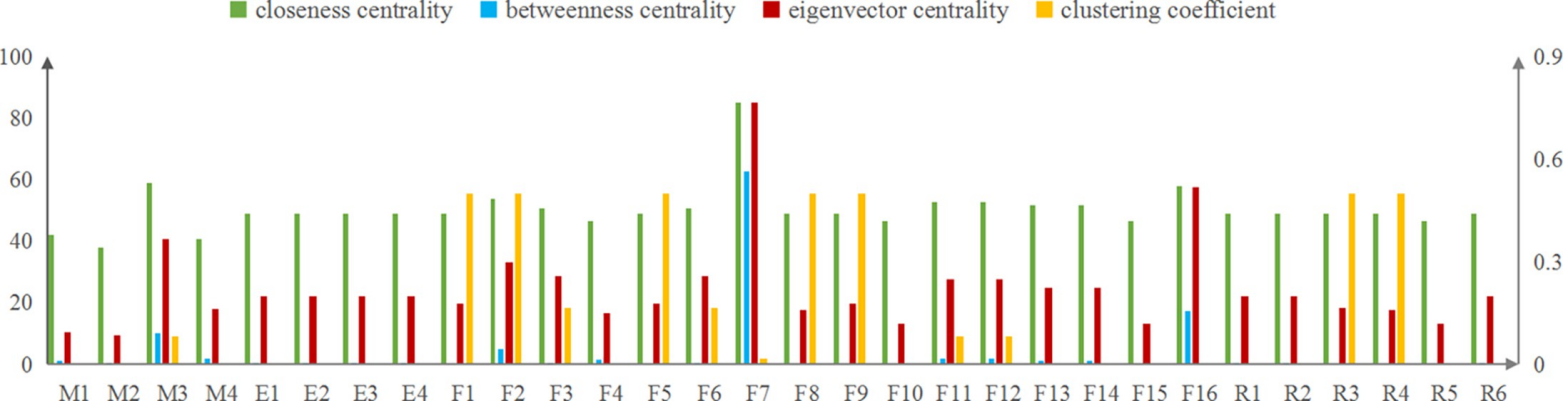
Analysis results of other node topology features in SVDRN.

Fig 5 demonstrates the distribution of closeness centrality, betweenness centrality, clustering coefficient and eigenvector centrality of 30 nodes in SVDRN. Except for a few nodes, there was little difference in the closeness centrality of nodes, and the mean value of closeness centrality was 50.302. The top three nodes of closeness centrality in the risk network were F7(improper operation), M3(inefficient driver management), and F16 (loss of vehicle control). From the global perspective of the network, these nodes located in the center of the SVDRN, and were close to other nodes, the result of which was not susceptible to be affected by other nodes and played a more obvious role in risk transmission.

The calculation result of the nodes in SVDRN showed that the betweenness centrality among nodes varied tremendously. The mean value of betweenness centrality was 3.654. Almost 30% of nodes had the zero value, so it was hard for them to be hubs in the shortest path between pairs of other nodes. The node of F7 (improper operation) with the largest value of betweenness centrality located in the core position of SVDRN, indicating that it was easier to affect the whole network than other nodes. The following nodes were F16 (loss of vehicle control), M3 (inefficient driver management), F2(speeding) and M4 (negligent vehicle technical management).

The clustering coefficient with the value of non-zero had 13 nodes in SVDRN, and the rest of 17 nodes were zero. According to the concept of the clustering coefficient, the clustering effect for nodes with only one neighbor node did not exist, such as R2 and R5. The values of clustering coefficient of seven nodes, including F1 (driving without a license), F2 (speeding), F5 (drunk driving), F8 (negligent observation), F9 (driving in the unapproved line), R3 (running over pedestrians), and R4 (fire), were relatively remarkable in Fig 4. The nodes of M3, F16 and F7 with low value are worthy of note, which could be explained that these three nodes had many neighbor nodes, but the probability of direct connection between neighbor nodes was low.

The mean values of eigenvector centrality in the SVDRN was 0.223. The node of F7 (improper operation) was the node with the highest eigenvector centrality at the value of 0.766. To sum up, improper operation ranked the first risk factor according to four indicators of topological features except for clustering coefficient. The following nodes with a higher value of eigenvector centrality were F16 (loss of vehicle control), M3 (inefficient driver management) and F2 (speeding). The four nodes were obtained in the light of three aspects: the amount of nodes connected to a node, the frequency acting as a intermediary node, and the importance ranking of neighbor nodes. The numeric value of eigenvector centrality for other nodes in the SVDRN were no more than 0.3.

### Evaluation result of comprehensive importance of network nodes

**Fig 6:**
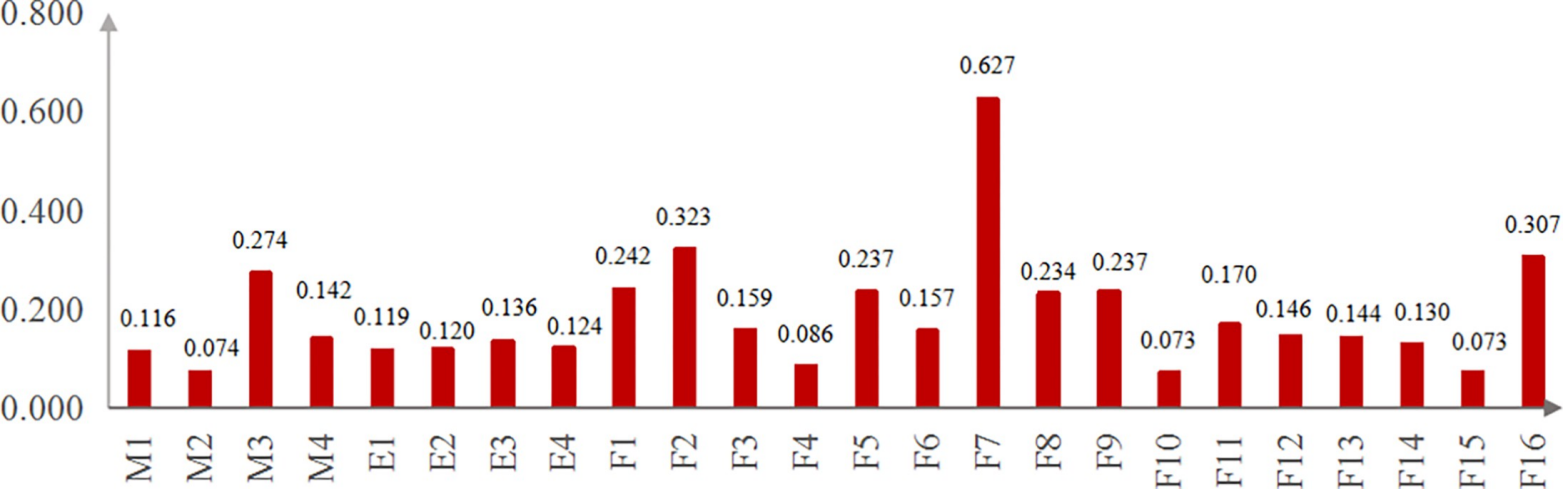
The comprehensive importance of nodes in SVDRN.

Fig 6 shows the comprehensive importance of nodes in the SVDRN calculated corresponding to Eqs (6)~(11), with the mean value of 0.185 and a standard deviation of 0.119. The larger its value was, the more core it was in the whole network. According to the foregoing evaluation model of the node importance, the order of influencing factors were listed from high to low as follows: (1) the node importance at the value of more than 0.3 for driver factors and vehicle factors: F7 (improper operation), F2 (speeding), F16 (loss of vehicle control), F1(driving without a license), F5 (drunk driving), F9 (driving in the unapproved line), F8 (negligent observation). These 7 nodes were also the top seven nodes importance in the whole risk network. (2) the importance sequence of management factors: M3(inefficient driver management), M4(negligent vehicle technical management), M1(vehicle ownership), and M2(failed vehicle dynamic monitoring); (3) the importance sequence of environmental factors: E3(non-standard road alignment), E4(unfavorable terrain), E2(inadequate road infrastructure), and E1(severe weather).

As can be shown from Fig 6, driver factors played a leading role in vehicle driving safety, especially the driver’s emergency decisions and behavior habits. When drivers encounter all kinds of emergencies, they need to make disposal decisions in a very short time. The speed and accuracy of driver’s response capability under emergency situation are the main influence factors for the occurrence and severity of traffic accidents [51]. The drivers’ response capability under emergency situation is mainly manifested in four aspects: psychological quality, physiological characteristics, decision-making ability and driving skills. The previous studies was more focused on the evaluation of drivers’ response capability, and found that the driver’s individual differences is significant [52, 53]. At present, the analysis of typical accident cases is mostly adopted to improve the stress response of professional drivers in the process of safety management of road transport enterprises. A few of transport enterprises use the driving simulator to conduct a certain number of danger scene training, but the management effect is insignificant because of not consistent with the real traffic conditions. In fact, the major accident is rarely triggered by a single factor.

The Apriori algorithm is used to analyze the combination correlation of four risk factors from Table 1. The analysis results show that the combination correlation of improper operation (F7) and non-standard road alignment (E3) has the highest, with the support of 51.81% and the confidence of 69.35%. In addition, the combination of speeding (F2) and improper operation (F7) has the second highest support rate at the value of 32.53%, and their confidence is 75.0%.

There were minor differences of 0.017 in the node importance among environmental factors, among which severe weather had the smaller value. However, the accident rate of severe weather (E1) was 31.33% in the single-vehicle accidents with 10+ fatalities. The accident rates of rainy, snowy and foggy days were 30.12%, 8.43% and 4.82%, respectively. A small number of the incidents involved two weather attributes, such as sleet or rain mixed with fog. The combination support of E1-F7 and E1-F2 is 32.53% and 27.71%, respectively.

### Results of risk chain identification

The monitoring practice of risk status for three passenger vehicles in a transport enterprise was carried out, so as to prove the scientific validity of the proposed method in this paper. The data came from a technical management database throughout the life cycle of vehicles. If the enterprise has not established an integrated database, the data for this study can also be obtained step-by-step on the premises that the enterprise has the active safety intelligent prevention and control system. The real-time dynamic information of test vehicle is applied to identify risk paths in combination with static daily management information. On the basis of data collection, the proposed algorithm for key risk chain identification was used to calculate the risk degree of all the risk chains. Table 3 demonstrates the analysis results of the risk chain analysis for the test vehicles, and the path set with the highest risk degree is output. The path length of risk chains displayed in Table 3 were short, which explained why single-vehicle accidents happened frequently, to a certain extent. For transport enterprises, the key risk chains of multiple vehicles are comprehensively analyzed to find out the high-frequency risk chains and risk factors. Combined with the comprehensive importance of their nodes, the enterprise can take effective measures before the accident. Any one or more causation nodes in the key risk chains can be removed, the loss of the accident will be reduced.

**Table 3:** Analysis result of risk chain.

Test vehicles	The path set with the highest risk degree	Path risk degree
1	*E2-F7-R4*	*L*(*24*,*1*) = 1.6×10^−3^
2	*E4-F16-R2*	*L*(*29*,*1*) = 2.6×10^−4^
3	*M2-F2-F7-R3*	*L*(*21*,*1*) = 7.9×10^−4^

## Conclusions

A comprehensive mode in this paper that integrated SDG model, hierarchical threshold, the multi-attribute comprehensive evaluation model and ergodic search algorithm was proposed to monitor the risk status of SVDRN and identify key risk chains. Among these, SDG model was used to construct the SVDRN, the multi-attribute comprehensive evaluation model were adopted to evaluate the node’s comprehensive importance, jointed with hierarchical threshold of node risk status for measuring the risk intensity of accident causation factors, three of which provided a basis for the algorithm of key risk chain recognition. This study provides novel insights into the identification of high-risk factors and paths for single-vehicle accidents and providing effective and specific countermeasures for mangers, especially when the prevention of major accidents is to be explored. With the gradual improvement of vehicle intelligent level for road transport enterprises, its active safety intelligent prevention and control system can be upgraded to realize the synchronous management of dynamic risk information and static daily management information for each vehicle on the monitoring platform. The model and algorithm proposed in this paper are embedded into the computing software to identify the high-risk risk chain and risk factors. The dynamic data of the active safety intelligent prevention and control system can ensure the real-time update. The change frequency of vehicle daily management data in Table 2 is lower, but they also needs to be updated regularly in the practice of enterprise safety management, so as to ensure the accuracy of risk assessment.

The SDG model of SVDRN and the rationality of evaluation indicators corresponding to risk factors need to be further refined, so that the data of safety management throughout the life cycle of vehicles is fully utilized. Moreover, due to significant differences in the risk mechanism of single- and multi-vehicle accidents, multi-vehicle driving risk network is a subject worth studying. Comparing the similarities and differences between them is of great reference value to the accident prevention in the process of road transportation. In consideration of complexity of risk coupling mechanism, the key risk chain recognition algorithm established in this paper needs further refinement, especially the computational method of path risk degree.

## Supporting information

S1 File Matrix M-data. (XLSX)

S2 File The data of Apriori algorithm. (TXT)

S3 File Apriori algorithm code. (TXT)
